# Two-step purification of elastin-like polypeptide-fusion superoxide dismutase via hydrophobicity and thermoresponsiveness

**DOI:** 10.3389/fbioe.2025.1695586

**Published:** 2025-10-24

**Authors:** Weiwei Wang, Jinping Chen, Hao Zhu, Aixia Huang, Dongren Zhou, Yuchen Wang, Yang Zhou, Feng Lin, Xiangai Dong, Yu Wu

**Affiliations:** ^1^ Key Laboratory of Healthy Freshwater Aquaculture, Ministry of Agriculture and Rural Affairs, Zhejiang Institute of Freshwater Fisheries, Huzhou, Zhejiang, China; ^2^ College of Biology and Food Engineering, Suzhou University, Suzhou, Anhui, China; ^3^ Nephrology Department, The Affiliated Xuzhou Municipal Hospital of Xuzhou Medical University, Xuzhou, China; ^4^ School of Life Sciences, Jiangsu University, Zhenjiang, Jiangsu, China

**Keywords:** superoxide dismutase, elastin-like polypeptide, hydrophobicity, foam separation, purification

## Abstract

**Introduction:**

Superoxide dismutase (SOD) catalyzes the dismutation of superoxide radicals to oxygen and hydrogen peroxide, serving as a key antioxidant enzyme with important therapeutic and industrial applications. However, the purification of recombinant SOD remains challenging due to low expression levels and the complexity of traditional purification methods, which involve time-consuming and multi-step chromatography. Elastin-like polypeptides (ELPs) offer a promising alternative due to their hydrophobic and thermoresponsive properties, which can be leveraged for non-chromatographic purification.

**Methods:**

A fusion protein of human SOD and an ELP tag (hSOD-ELP) was produced. The purification strategy consisted of two sequential steps. First, foam separation was employed, exploiting the hydrophobicity of the ELP to selectively adsorb hSOD-ELP at the gas-liquid interface. Second, inverse transition cycling (ITC) was used to further purify hSOD-ELP by exploiting ELP’s thermoresponsiveness.

**Results:**

Under optimized conditions (0.4 mg/mL protein, 30 °C), the initial foam separation step achieved an enrichment ratio of 1.93, a protein recovery of 85.67%, an enzyme activity enrichment of 2.15, and an activity recovery of 93.32%. The subsequent ITC step yielded a recovery rate of 91.98% and a purification fold of 17.45. The cumulative two-step process resulted in a total yield of 85.84% and overall purification fold of 37.52, yielding the purified hSOD-ELP with a final purity of approximately 85%.

**Discussion:**

These results demonstrate that ELP-mediated purification offers a scalable and economical alternative to conventional methods. The combination of foam separation and thermal precipitation minimizes the need for expensive chromatography, making this strategy particularly promising for industrial-scale biotechnological applications.

## 1 Introduction

Foam separation is an emerging separation technology that relies on the selective adsorption of substances at the gas-liquid interface. Its underlying principle is that surface-active materials have a propensity to accumulate at the interface, enabling the enrichment and separation of specific components. This technique has gained substantial attention in various fields, especially in the separation of proteins (Fang et al., 2023; Wang et al., 2023). Proteins, being amphiphilic molecules with both hydrophilic and hydrophobic groups, have a natural tendency to adsorb at the gas-liquid interface. This characteristic makes them suitable for separation via foam flotation. The process typically involves introducing air into a protein solution, forming bubbles that selectively adsorb proteins. These protein-laden bubbles subsequently rise to the surface, forming a foam layer that can be collected and further processed. This method is particularly effective for the concentration and recovery of proteins from dilute solutions, offering a low cost, easy operation, no pollution, and high efficiency alternative to traditional separation techniques.

Xu et al. employed foam fractionation to enhance the silica gel adsorption (SGA) of urokinase from human urine. They investigated the impacts of pH, superficial air flow rate, beta-cyclodextrin concentration, and the amount of silica gel added on the activity recovery yield and purification fold of urokinase (Xu et al., 2019). The activity recovery and purification fold of urokinase reached 89.5% and 56.8, respectively, which were 25.3% and 79.2% higher than those obtained by SGA alone (Xu et al., 2019). Tian et al. developedd a method combining foam fractionation with isoelectric precipitation to effectively recover casein from its highly diluted solution (Tian et al., 2018). Then, the foamate was treated with precipitation, and the supernatant could be reused as the feed solution for the first-stage foam fractionation. As a result, the enrichment ratio was further increased to 52.6, and the recovery percentage reached 91.7%. In two-stage continuous foam fractionation, the enrichment ratio and recovery percentage of casein were obtained as high as 12.1% and 92.3%, respectively (Tian et al., 2018). Li et al. utilized Na-citrate to enhance the self-association of bovine serum albumin (BSA) at the gas–liquid interface and improve the stability of protein foams (Li et al., 2016). Under optimal conditions, the enrichment ratio and recovery of BSA were 14.6% and 57.6%, respectively (Li et al., 2016).

Superoxide dismutase (SOD) is a crucial antioxidant enzyme that catalyzes the dismutation of superoxide radicals (O_2_^−^) into oxygen and hydrogen peroxide, thereby protecting cells from oxidative damage (Saxena et al., 2022). Consequently, SOD has garnered significant attention due to its potential anti-aging, antiviral, and anti-inflammatory effects in living organisms (Chidambaram et al., 2024). SOD from various organisms have been cloned, heterologously expressed and purified. The purification of SOD is essential for its applications in pharmaceuticals, cosmetics, and food industries. Traditional purification methods, such as affinity chromatography or ion-exchange chromatography, are among the most versatile and powerful techniques for isolating specific molecules or groups of molecules from complex mixtures. However, these methods have several drawbacks. The high cost of affinity resins, yield losses during purification, and reduced activity of the target protein due to multiple washing steps are major challenges. Additionally, in some cases, the presence of a large quantity of impurities can further complicate the purification process. Therefore, there is a need to explore more cost-effective and efficient purification strategies to improve the yield while minimizing impurities.

In recent years, alternative approaches leveraging the unique properties of fusion tags have been explored to simplify and enhance the purification process. Elastin-like polypeptide (ELP) are synthetic, thermally responsive polypeptides composed of repeating pentapeptide sequences with the general formula (VPGXG)n; where X can be any amino acid except proline, and n represents the number of repeats, typically ranging from 20 to 330 (Han et al., 2022; Meyer and Chilkoti, 2002). ELP exhibits a quick and thermodynamic reversible phase transition behavior at a specific temperature referred to as the inverse transition temperature (Tt). Below their Tt, ELP are structurally disordered and soluble in aqueous solution. Conversely, when the temperature exceeds Tt, the ELP becomes insoluble and begins to aggregate, which can then be easily separated by centrifugation (Addai et al., 2025; Sugawara-Narutaki, 2025). When ELPs are genetically fused to a target protein, the resulting ELP-fusion protein retains the characteristic inverse transition behavior (Chen et al., 2023). This behavior allows for a simple method to isolate a recombinant ELP fusion protein from cell contaminants by taking the solution through the soluble and insoluble phase of the ELP fusion protein, a technique designated as the inverse transition cycling (ITC) (Sugawara-Narutaki, 2025). This property enables a straightforward and efficient purification strategy, facilitating the isolation of recombinant ELP-fusion proteins from cell contaminants (Shin and Chae, 2024). Several studies have demonstrated the successful purification of recombinant enzymes using ELP fusion tags. We previously reported the purification of β-galactosidase e using ELP fusion, achieving a purification fold of 13.04 and a recovery rate of 95.66% (Peprah Addai et al., 2020). Similarly, Wang et al. utilized ELP fusion to purify endoglucanase, resulting in a purification fold of 11.8 and a recovery rate of 78.1%. Furhermore, The ELP fusion endoglucanase had a better thermostability, higher optimal temperature, and longer half-life than those of free endoglucanase (Wang et al., 2020b). These studies highlight the efficiency and simplicity of the ELP-based purification approach.

In our previous study, ELP was fused to human superoxide dismutase 1 (hSOD) modified with His tag to produce recombinant hSOD-Linker-ELP-His (hSODLEH) which was expressed in *Escherichia coli* and purified via ITC and Ni-NTA resin. The results showed that the purification by ITC was superior to Ni-NTA resin due to its convenient purification process, improved recovery rate and purification fold (Wang et al., 2024). In this study, we presents a two-step purification strategy for recombinant hSODLEH. The first step involves foam separation, leveraging the hydrophobicity of the ELP tag to selectively adsorb hSODLEH at the gas-liquid interface. The second step utilizes ITC to further purify hSODLEH. The total yield and purification fold was 85.84% and 37.52 after the two-step purification, yielding the purified hSODLEH with a final purity of approximately 85%.

Our study integrates foam separation with the thermoresponsive and hydrophobic properties of ELP, offering a more efficient and cost-effective purification process compared to traditional methods. Additionally, we provide insights into the aggregation behavior and surface hydrophobicity of hSODLEH, which contribute to its enhanced adsorption and separation efficiency. These findings not only demonstrate the potential of ELP-mediated strategies for protein purification but also highlight the versatility of combining different physical properties for improved biotechnological applications.

## 2 Materials and methods

### 2.1 Materials

The expression vector pET-28a (+) and *E. coli* strains DH5a and BL21 (DE3) were stored in our lab. Kanamycin, protease inhibitors phenylmethanesulfonyl fluoride (PMSF), isopropyl β-D-1-thiogalactopyranoside (IPTG), Glycine, Tris, SDS, Bromophenol blue and Coomassie brilliant blue R 250 were purchased from Sangon Biotech (Shanghai, China). Thermo Scientific Pierce BCA Protein Assay Kit was purchased from Thermo Fisher Scientific (MA, United States). CuZn-SOD activity assay kit (WST-8 method) was purchased from Beyotime (Shanghai, China). Isopropanol, absolute ethanol, β-mercaptoethanol, ammonium persulfate, 1-ethyl-(3-dimethylaminopropyl) carbodiimide hydrochloride (EDC), N-hydroxysuccinimide (NHS) and glacial acetic acid were obtained from Sinopharm Chemical Reagent (Shanghai, China). Ammonium 8-phenylamino-1-naphthalene sulfonate (ANS) was purchased from Merrier Laboratory Equipment Co., Ltd. (Shanghai, China). Fluorescein isothiocyanate (FITC) was purchased from Maclin Biochemical Technology Co., Ltd. (Shanghai, China). Ni-NTA Resin was purchased from GenScript (Nanjing, China). All chemical reagents were of analytical grade and were used without further treatment.

### 2.2 Construction of recombinant expression plasmids

The amino acid coding sequence for hSOD were obtained from GenBank (accession number: CR541742.1). The hSOD gene was fused with a 50-repeating pentapeptide ELP sequence ((VPGVG)_50_) via a flexible linker (GGGGS)_3_. The resulting nucleotide sequence was synthesized by Synbio Tech (Jiangsu, China) and subcloned into the pET28a (+) vector to generate the recombinant expression plasmid pET28a (+)-hSODLEH (hSOD-Linker-ELP-6xHis, hSODLEH). Additionally, a recombinant plasmid pET28a (+)-hSOD containing the hSOD gene and a 6xHis-tag was constructed.

The pasmids (pET28a (+)-hSODLEH, pET28a (+)-hSOD, or empty vector) are transformed into *E. coli* BL21 (DE3) for protein production. Briefly, a single colony was incubated overnight in LB medium with kanamycin (50 μg/mL) with shaking at 37 °C. The overnight culture was then diluted 1:100 into 1 L of fresh LB medium (containing kanamycin) and grown at 37 °C with shaking. When OD_600_ reached 0.4–0.6, IPTG was added to a final concentration of 0.2 mM to induce protein expression at 25 °C with shaking at 200 rpm. After shaking about 6 h, the cells were harvested by centrifugation at 4,500 g, and the cell pellets were resuspended in Tris-HCl buffer (50 mM, pH8.0). After adding PMSF, the cells was lysed by sonication (ultrasonic disruption) for 30 min, with alternately sonication 10 s and intermittent periods of cooling 10 s. The lysate was centrifuged at 12,000 × g, 4 °C, 20 min twice to separate soluble (supernatant) and insoluble (pellet) fractions. For detailed procedures regarding the construction and expression, please refer to our previous studies (Lin et al., 2018; Wang et al., 2024).

### 2.3 Foam separation

**FIGURE 1:**
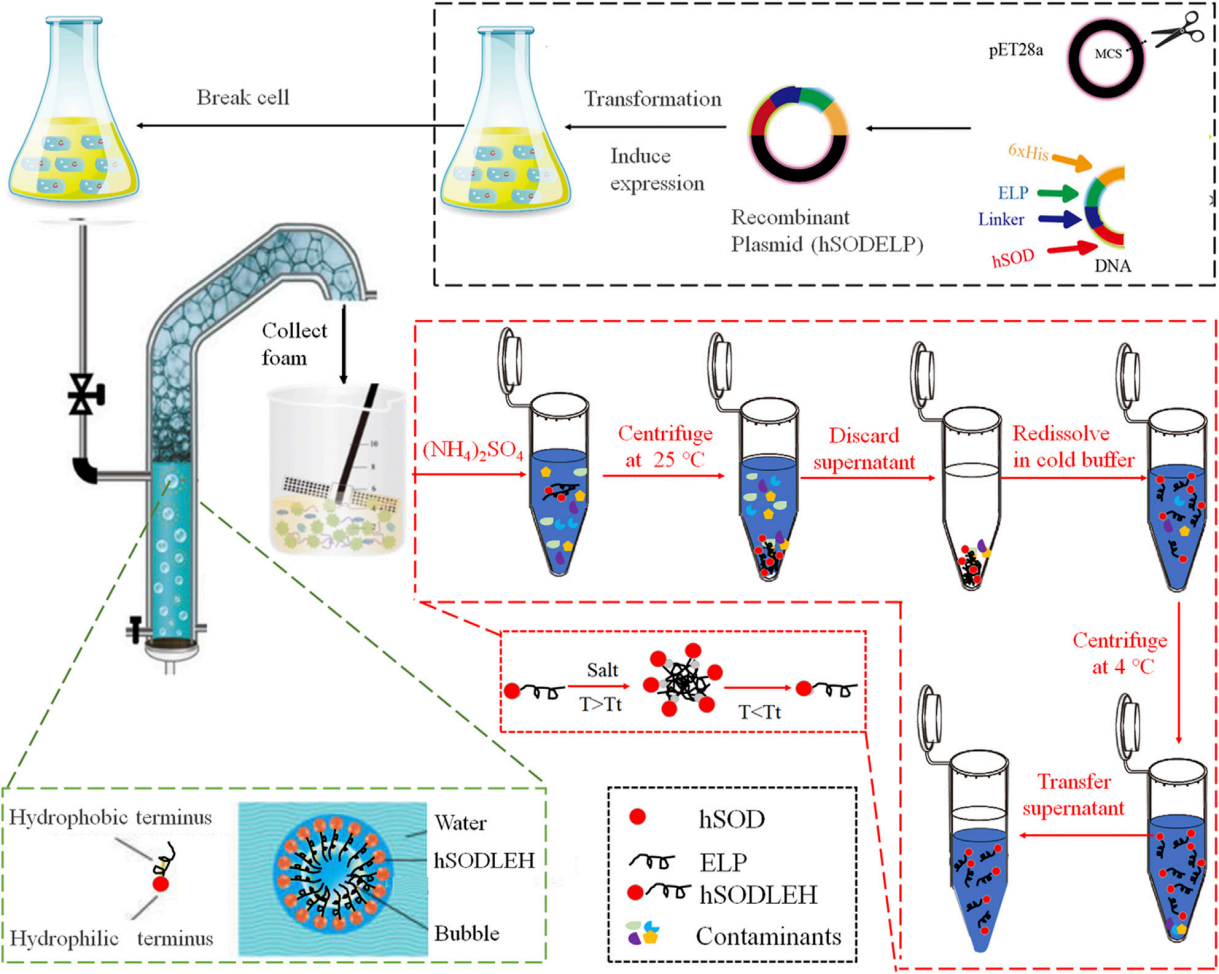
Schematic diagram of foam separation and further ITC purification of hSODLEH.

The foam separation equipment was designed by our lab (Fang et al., 2023; Xue et al., 2024). The process of foam separation and purification of hSODLEH is shown in Figure 1. The cell lysis solution of hSODLEH or hSOD, with different concentrations (0.2–1.0 mg/mL) was added to the separation column. The temperature was controlled using a circulating water bath device, maintained at 25, 30, 35, 40, and 45 °C. Nitrogen was passed into the separation column at a gas flow rate of 300 mL/min and a gas flow time of 22 s. Foam was collected until it no longer flowed out of the tower mouth. Mechanical agitation was employed to defoam the collected foam, yielding the final defoamed solution.

The foam separation efficiency of recombinant enzyme was evaluated by the enrichment ratio of the protein (Ep) (Equation 1), the protein recovery percentage (Rp) (Equation 2), the enrichment ratio of enzyme activity (Ee) (Equation 3), and the enzyme activity recovery percentage (Re) (Equation 4).

$$E_{p}=\frac{C_{b}}{C_{i}}$$ (1)

$$R_{p}=\frac{C_{b}\times V_{b}}{C_{i}\times V_{i}}\times 100\%$$ (2)

$$E_{e}=\frac{U_{b}}{U_{i}}$$ (3)

$$R_{e}=\frac{{C_{b}\times V_{b}\times U}_{b}}{C_{i}\times V_{i}\times U_{i}}\times 100\%$$ (4)

Where *C*_*b*_ indicates the protein concentration of defoaming solution (mg/mL). *C*_*i*_ is the initial protein concentration (mg/mL). *V*_*b*_ represents defoaming liquid volume (mL). *V*_*p*_ is the liquid intake volume (mL). *U*_*b*_ indicates specific enzyme activity of defoaming solution (U/mg). *U*_*i*_ represents the initial specific enzyme activity (U/mg).

### 2.4 ITC purification

hSODLEH was further purified using ITC, according to previous experiments in our lab (Addai et al., 2025; Peprah Addai et al., 2020). Initially, to determine the optimal salt for purification, different salts, namely, 1 M NaH_2_PO_4_, 1 M (NH_4_)_2_SO_4_, 1 M Na_2_SO_4_, and 2.5 M NaCl, were employed to purify hSODLEH. Following SDS–PAGE analysis, (NH_4_)_2_SO_4_ was identified as the most suitable purification salt. Subsequently, a certain amount of (NH_4_)_2_SO_4_ was added into the cell lysis solution of hSODLEH. The mixed solution was incubated at 25 °C for 20 min and then centrifuged at 25 °C, 12,000 rpm for 10 min. The supernatant was discarded and the pellet resuspended in cold Tris-HCl buffer (50 mM, pH 8.0). The samples were incubated in an ice water for 1 h, and then centrifuged at 12,000 rpm, 4 °C for 20 min. The supernatant was the purified hSODLEH.

### 2.5 Enzyme activity assay

The catalyzing activity of the recombinant hSODLEH or hSOD was measured by Cu/Zn-SOD assay kit. Following the manufacturer’s instructions, a preliminary experiment was performed to determine the optimal amount of hSODLEH or hSOD required to achieve an inhibition rate between 30% and 70%. The enzyme working solution (160 μL), certain amount of hSODLEH or hSOD (20 μL) and reaction starting solution (20 μL) were incubated for 30 min at 37 °C in a thermostatic water bath (Honghua, Jiangsu, China) under static conditions. The absorbance was then measured at 450 nm using Synergy H4 hybrid multi-mode microplate reader (BioTek, United States). The activity of the enzyme was calculated in accordance with the instructions provided by the manufacturer.

### 2.6 Dynamic light scattering (DLS)

The particle size distribution in hSODLEH or hSOD solutions was analyzed using a laser particle size analyzer (Litesizer ™ 500, Anton Paar, Austria). One mL of hSODLEH or hSOD (1 mg/mL) was transferred to a 1.5 mL Eppendorf tube. (NH_4_)_2_SO_4_ was added to achieve a final concentration of 200 mM, and the mixture was vortexed thoroughly. The mixture was loaded into a quartz cuvette, and DLS measurements were carried out at 4 °C (<Tt) or 55 °C (>Tt), with an equilibration time of 5 min. The spectrogram data were collected.

### 2.7 Fluorescence labeling

Ten mL (1 mg/mL) of either the hSODLEH or hSOD solution was reconstituted in sodium acetate buffer (50 Mm, pH 5.5). Then, 5 mg of EDC and 2.5 mg of NHS were added to the enzyme solution and stirred at 4 °C for 60 min. A total of 63 μg of FITC was added to the enzyme-buffer mixture. The resulting solution was then stirred gently in darkness at 4 °C for 2 h. The fluorescence-labeled enzyme were dialyzed at 4 °C for 24 h in darkness to remove residual FITC, EDC and NHS. After the dialysis, a final concentration of 200 mM (NH_4_)_2_SO_4_ was added to the enzyme solution. The mixture was incubated for 30 min at either 4 °C (<Tt) or 55 °C (>Tt). Fluorescence was measured at 519 nm after excitation at 488 nm using confocal laser microscopy (Leica TCS SP5, Leica instrument).

### 2.8 Determination of surface hydrophobicity

The surface hydrophobicity of hSODLEH and hSOD was determined using the fluorescent probe ANS. The 1 mg/mL hSODLEH or hSOD was stepwise diluted with Tris-HCl (50 mM, pH8.0) to concentrations of 0.1, 0.3, and 0.5 mg/mL. The mixtures were homogenized and incubated at 4 or 55 °C for 30 min. The ANS solution (8.0 mM) was added to the solutions to achieve a final concentration of 400 µM. After full mixing, the solutions were equilibrated at room temperature for 1 h. 300 μL was transferred to a quartz sample cell. The fluorescence intensity was measured, starting from the lowest to the highest concentration, using a spectrofluorometer (Agilent Technologies, Australia), with excitation and emission slits set at 5 nm, excitation wavelength of 380 nm and emission wavelength in the range of 420–580 nm at a rate of 800 nm/min. Each concentration was repeated three times. The relative fluorescence intensity of protein dilution blanks (no ANS) and a buffer blank (buffer + ANS) were also measured. The relative fluorescence intensity of each protein dilution blank was subtracted from that of the corresponding protein dilution with ANS to provide net relative fluorescence intensity. Standardization of net relative fluorescence intensity values was based on measuring the relative fluorescence intensity for ANS (10 µL) in methanol (10 mL) and normalizing to a standard value of 70 (Glibowski et al., 2006; Paraman et al., 2007). Surface hydrophobicity (S_0_) was expressed as the initial slope of the plot of standardized net relative fluorescence intensity values vs. % protein concentration.

## 3 Results

### 3.1 Construction, expression of ELP-tagged recombinant protein (hSODLEH)

We previously successfully constructed the plasmids for hSOD and hSODLEH, with the nucleotide fragment lengths being 462 bp for hSOD and 1,293 bp for hSODLEH. These recombinant plasmids were successfully expressed in *E. coli* BL21 (DE3) cells, and the expressed proteins were predominantly in a soluble form (Wang et al., 2024). The apparent molecular weights of the expressed proteins were approximately 20 kDa for hSOD and 40 kDa for hSODLEH (Wang et al., 2024).

### 3.2 Size distribution and aggregation of hSODLEH

The Tt of the hSODLEH was determined in our previous study. Results showed that when the protein concentration was 0.5 mg/mL, and 0, 100, 200, 300, 400 and 500 mM of (NH_4_)_2_SO_4_ was added, the Tt was >80, 77.3, 60.0, 31.3, 27.0 and <22 °C, respectively. When the concentration of hSODLEH increased to 1 mg/mL, the Tt of hSODLEH were observed to be >80, 56.0, 43.7, 28.7, <22 and <22 °C (Wang et al., 2024). To investigate the aggregation behavior characteristics, DLS and confocal fluorescence microscopy imaging were used to characterize at 1.0 mg/mL hSOD and hSODLEH in the presence of 200 mM (NH_4_)_2_SO_4_ at below the Tt (4 °C) or above the Tt (55 °C).

**FIGURE 2:**
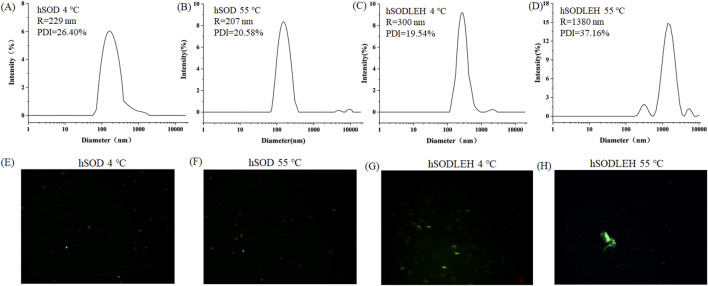
DLS curves **(A–D)** and fluorescent fields **(E–H)** of the hSOD and hSODLEH at different temperatures (4 or 55 °C).

The hydrodynamic radius (Rh) of hSOD and hSODLEH at 4 °C were 229 and 300 nm, respectively (Figures 2A,C). This indicated that both hSOD and hSODLEH primarily existed as monomers dispersed uniformly in the solution, with relatively even distribution among particles. When the temperature was increased to 55 °C, the average Rh values for the hSODLEH increased significantly to 1,380 nm (Figure 2D). However, the average Rh value for hSOD did not increase significantly and remained at 207 nm (Figure 2B). This suggested that hSODLEH self-assembled into larger aggregates at 55 °C, while hSOD did not exhibit significant aggregation under the same conditions.

Fluorescence confocal microscopy analysis was performed to further characterize aggregate behavior at 4 or 55 °C. The microscopy images revealed that at 4 °C, both hSOD and hSODLEH were uniformly dispersed (Figures 2E,G). However, at 55 °C, hSODLEH underwent self-aggregation, forming large protein aggregates (Figure 2H). This demonstrated that hSODLEH could form larger protein aggregates through self-aggregation in the aqueous phase with the temperature increase, and the particle size also increased significantly.

### 3.3 Surface hydrophobicity of the hSOD and hSODLEH

**FIGURE 3:**
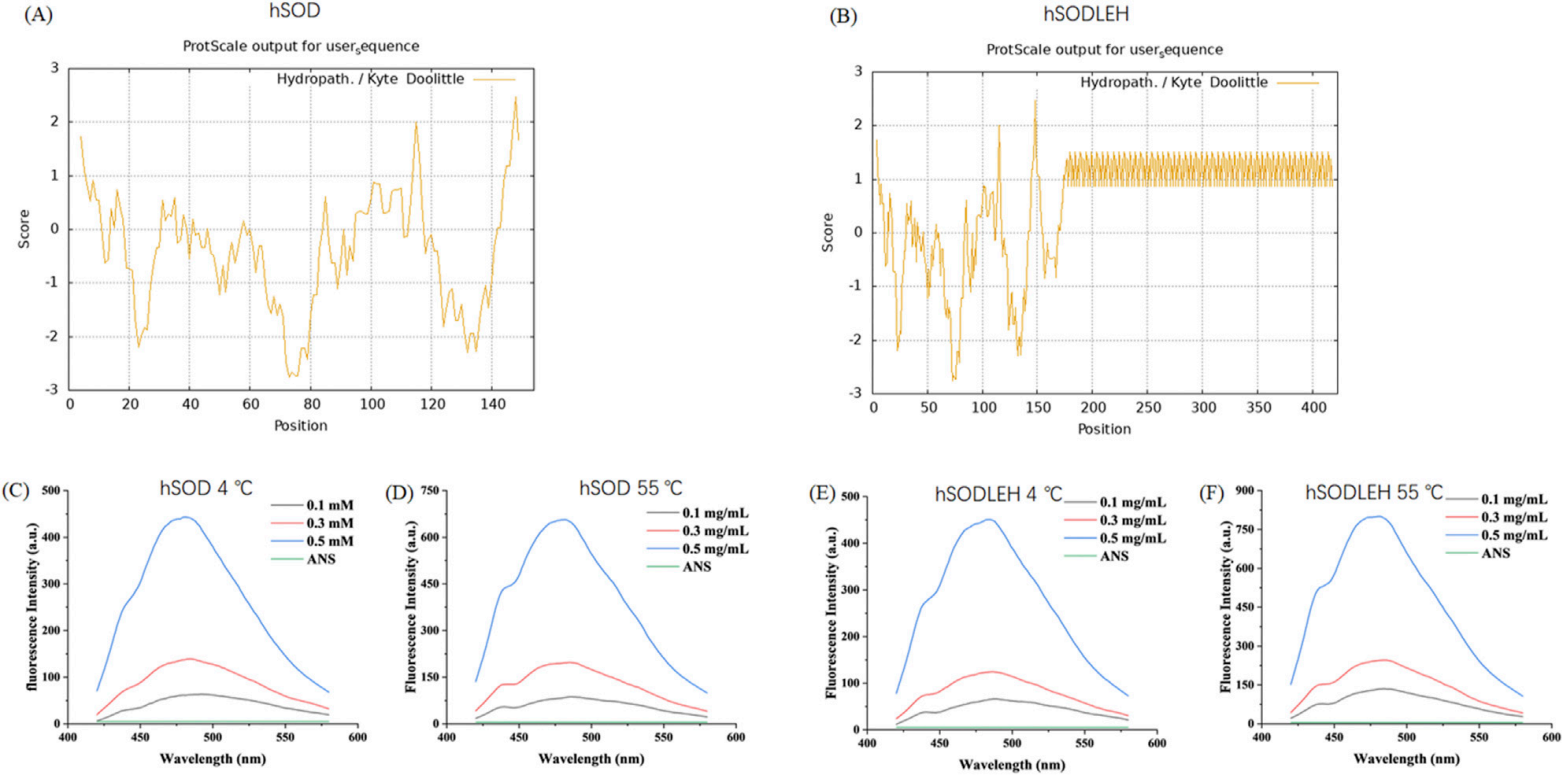
Hydrophobicity analysis of hSOD and hSODLEH **(A,B)**. Fluorescence intensity of hSOD and hSODLEH at 4 and 55 °C **(C–F)**.

To further investigate the temperature dependent aggregation mechanism of hSODLEH, we used the ProtScale online software (https://web.expasy.org/protscale) to predict the hydrophobicity of the protein. The larger the positive value stands for the more hydrophobic, and the larger the negative value means the more hydrophilic (Han et al., 2019; Zhang et al., 2021). The grand average of hydropathicity (GRAVY) (GRAVY) of hSOD and hSODLEH were −0.344 and 0.565, respectively, indicating that hSODLEH exhibits significantly higher hydrophobicity than hSOD (Figures 3A,B).

Addationally, the hydrophobic fluorescent probe ANS was utilized to explore the influence of external temperature on the surface hydrophobicity of hSOD and hSODLEH. ANS exhibits fluorescence in aqueous solution, however, its fluorescence intensity significantly increases upon binding to proteins, making it widely used for determining protein surface hydrophobicity. When the excitation wavelength was 380 nm, ANS had a maximum emission wavelength of around 520 nm when it existed alone, with a very low fluorescence intensity value of 5.03.

Upon the addition of hSOD and hSODLEH, the maximum emission wavelengths of ANS underwent a blue-shift to around 480 nm (Figures 3C–F). The surface hydrophobicity index S_0_ of hSOD and hSODLEH was illustrated in Supplementary Figure S1, and the value of S_0_ was shown in Table 1. At 4 °C, the S_0_ value of hSODLEH/ANS (1,054.60) was slightly higher than that of hSOD/ANS (935.90), indicating a moderate increase in hydrophobic exposure. When the temperature increased to 55 °C, the S_0_ value of hSODLEH/ANS rose significantly 1763.54, while hSOD/ANS showed a smaller increase to 1,384.80. This indicated that hSODLEH exposes more hydrophobic regions at elevated temperatures, consistent with its thermoresponsive aggregation behavior.

**TABLE 1:** Surface hydrophobicity indices (S0) of hSOD and hSODLEH at different temperatures.

Protein	Temperature (°C)	Surface hydrophobicity index (S_0_)	*R* ^2^
hSOD	4	935.90 ± 21.3	0.9987
hSOD	55	1,384.80 ± 32.5	0.9989
hSODLEH	4	1,054.60 ± 18.7	0.9997
hSODLEH	55	1,763.54 ± 25.1	0.9999

### 3.4 Effect of protein concentration on the foams separation of hSOD and hSODLEH

**FIGURE 4:**
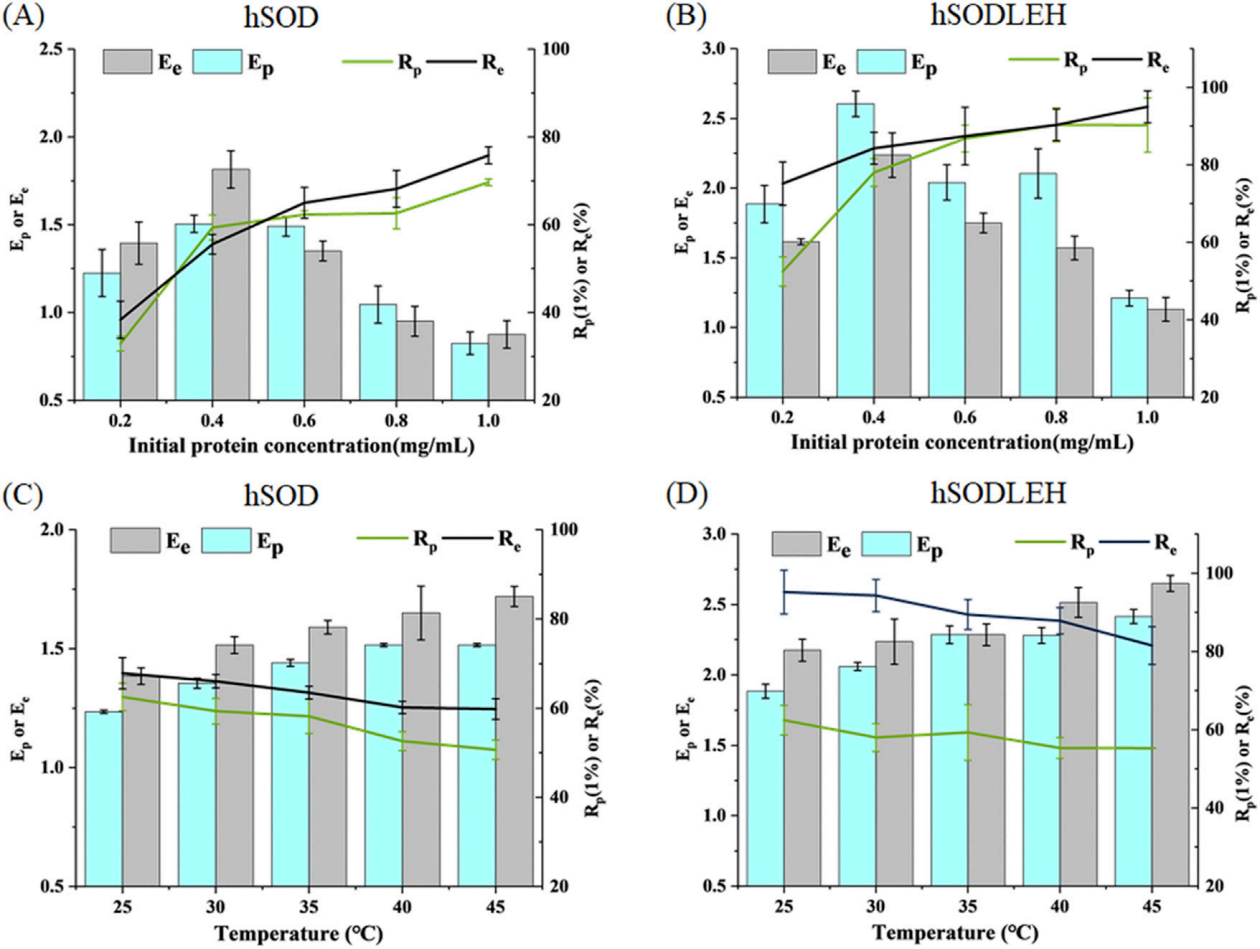
Effects of initial protein concentration **(A,B)** and temperature **(C,D)** of foam separation on hSOD and hSODLEH.

The influence of initial protein concentration of hSOD and hSODLEH cell lysis solution on foam separation was investigated, and the results are presented in Figures 4A,B. As the initial protein concentration increased, the recovery rates of both hSOD and hSODLEH increased, while the enrichment ratios decreased. Considering Ep, Rp, Ee, Re as evaluation criteria, initial protein concentrations of 0.4 mg/mL were selected as the appropriate conditions for subsequent experiments with both proteins.

The effect of temperature on the enrichment ratio and recovery percentage is shown in Figures 4C,D. As temperature increased from 25 °C to 45 °C, the enrichment ratio increased, while the recovery percentage decreased with increasing temperature. Balancing recovery percentage and enrichment ratio, 30 °C was chosen as the optimal temperature for performing foam separation.

In summary, the optimized foam separation conditions for hSOD and hSODLEH from cell lysis solution were determined as follows: the initial protein concentration of 0.4 mg/mL and the temperature of 30 °C.

### 3.5 Foam separation of hSOD and hSODLEH

Under the optimal conditions, the purification efficiency of foam separation of hSOD and hSODLEH were presented in Table 2. For hSODLEH, the values for Ep, Ee, Rp and Re were 1.93, 2.15, 86.57%, and 100%, respectively. For hSOD, the corresponding values of Ep, Ee, Rp and Re were 1.27, 1.36, 64.76%, and 65.49%, respectively. Notably, SOD activity was barely detectable in the residual liquid of the hSODLEH crude lysate after foam separation. In contrast, 34.50% of enzyme activity remained in residual liquid of hSOD after foam separation. These results indicated that ELP tag facilitates the specific adsorption of the recombinant enzyme onto the bubble surface, enabling the preferential separation and enrichment of the recombinant enzyme under these conditions.

**TABLE 2:** Effect of purification of hSOD and hSODLEH by Foam separation method under optimal conditions.

Enzyme	Fraction	Total protein (mg)	Total activity (U)	Ep	R_p_ (%)	Ee	Re (%)
hSOD	Foamate	42.13 ± 0.48	171.05 ± 0.81	1.27 ± 0.042	64.76 ± 0.97	1.36 ± 0.041	65.49 ± 0.51
	Residue	22.93 ± 0.24	90.11 ± 0.52	0.67 ± 0.027	35.24 ± 0.63	0.51 ± 0.025	34.50 ± 0.22
hSODLEH	Foamate	58.79 ± 0.36	340.39 ± 1.13	1.93 ± 0.034	86.57 ± 1.04	2.15 ± 0.087	93.32 ± 0.78
	Residue	9.12 ± 0.21	0	0.29 ± 0.009	11.77 ± 0.32	0	0

### 3.6 Further purification of hSODLEH by the thermally responsive property of ELP

**FIGURE 5:**
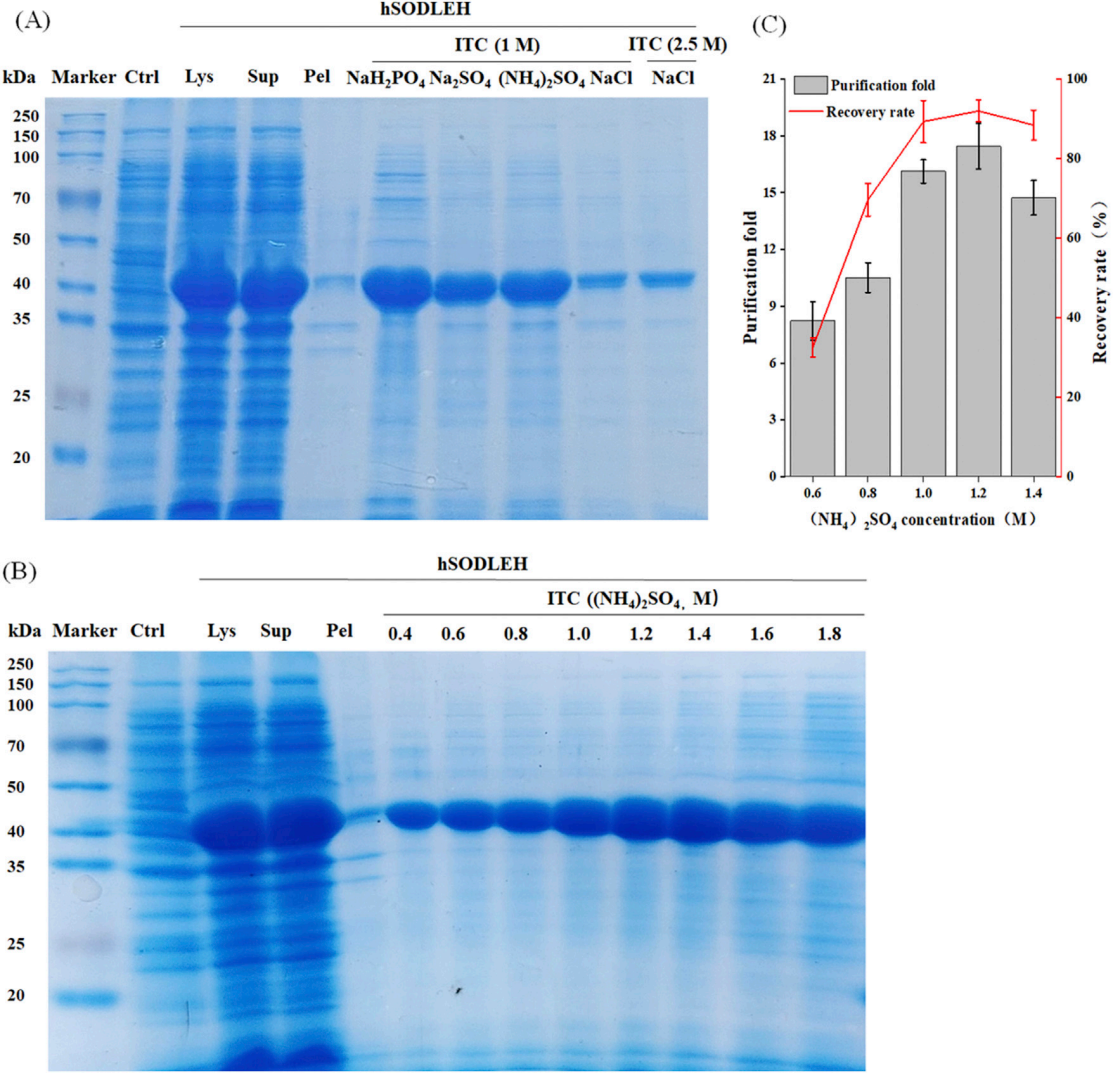
Optimization conditions for purification of hSODLEH by ITC. **(A)** SDS-PAGE analysis of hSODLEH purified by different salt ions (1 M NaH_2_PO_4_, 1 M Na_2_SO_4_, 1 M (NH_4_)_2_SO_4_, and 1 and 2.5 M NaCl). **(B)** SDS–PAGE of hSODLEH purified by ITC using 0.4–1.8 M (NH_4_)_2_SO_4_. **(C)** Recovery rate and purification fold of hSODLEH at different concentrations of (NH_4_)_2_SO_4_. Lane M–protein marker, Lane Ctrl, *E. coli* transformed with empty plasmid, Lanes Lys, Sup and Pel refers to whole cell lysate, supernatant and pellet of *E. coli* transformed with plasmid containing the expressing hSODLEH.

The hSODLEH was further purified using the thermally responsive property of ELP, with the defoaming solution fromfoam separation as the raw material. First, different types of salts on the purification efficiency of HSODLEH by ITC was investigated. Figure 5A showed that (NH_4_)_2_SO_4_ was the optimal salt. Secondly, various concentrations of (NH_4_)_2_SO_4_ on the purification efficiency of hSODLEH was test. Figure 5B showed that a clear band at around 40 kDa appeared, indicating that 0.4–1.8 M (NH_4_)_2_SO_4_ could be used to purify hSODLEH by ITC. However, as the concentration of (NH_4_)_2_SO_4_ increased from 1.0 to 1.8 M, more and more impurities appeared in the bands. According to Figure 5C, the purification fold of hSODLEH reached a maximum of 17.45, and the recovery rate was 91.98% at an (NH_4_)_2_SO_4_ concentration of 1.2 M. Nevertheless, considering various factors, 1.2 M (NH_4_)_2_SO_4_ was ultimately selected as the optimal purification salt for hSODLEH. hSOD without the ELP tag exhibited negligible aggregation and purification under identical conditions (data not shown), confirming that the thermoresponsive property of the ELP tag is essential for efficient ITC purification of hSODLEH. Overall, the total yield and purification fold after two-step purification was 85.84% (93.32% foam separation × 91.98% ITC) and 37.52 (2.15 foam separation × 17.45 ITC), respectively. Following the two-step purification process, the final purity of hSODLEH was approximately 85%, as determined by SDS-PAGE densitometric analysis (Figure 5B), with an absolute yield of approximately 8 mg per liter of *E. coli* culture.

## 4 Discussion

In the field of protein purification, it is crucial to develop efficient and cost - effective separation techniques for various biotechnological and pharmaceutical applications (Zhang et al., 2022). This study aimed to the effective separation and purification of hSODLEH from a cell lysis solution. The approach combines foam separation with an ELP fusion tag, leveraging their unique properties for enhanced purification efficiency.

ELP has emerged as a powerful tool for protein engineering and purification due to their unique thermal responsiveness and biocompatibility (Zhou et al., 2019). First, ELP exhibits a thermodynamic reversible phase transition behavior at Tt, enabling selective precipitation of the fusion protein from solution upon temperature change. This property simplifies the purification process, as the ELP-tagged protein can be easily separated from cell lysates and other contaminants by adjusting the temperature. Second, ELP are composed of repeating pentapeptide sequences that are biologically inert and do not significantly interfere with the structure or function of the target protein (Chilkoti et al., 2006). We have previously constructed several ELP-fusion proteins. Results demonstrated that the purification by ITC was superior to the traditional Ni-NTA resin. Moreover, the ELP did not affect the enzyme activity, kinetic parameters and secondary structure of ELP-fusion protein. More importantly, ELP improved the stability in harsh conditions of ELP-fusion protein such as heating and exposure to denaturant (Peprah Addai et al., 2020; Wang et al., 2024; Zhou et al., 2019). Finally, ELP is biocompatible, non-immunogenic, and can enhance the stability of fused proteins (Shi et al., 2022).

The surface hydrophobicity of ELP-tagged proteins is inherently associated with their phase transition behavior (Fang et al., 2023). In this study, hSODLEH had a positive GRAVY score (0.565), contrasting with hSOD’s negative score (−0.344), thereby validating the role of the ELP tag in enhancing hydrophobicity. This finding was consistent with the S_0._ The hydrophobic fluorescent probe ANS was employed to effectively visualize these changes. Below Tt (4 °C), the fluorescence enhancement of hSODLEH/ANS was slightly higher than that of hSOD/ANS. Above Tt (55 °C), However, hSODLEH/ANS exhibited significantly greater fluorescence enhancement, indicating that hSODLEH exposes more hydrophobic regions at elevated temperatures.

The increase in surface hydrophobicity is primarily attributed to the conformational change of the ELP tag. As the temperature exceeds Tt, ELP transform from a random, disordered structure to an ordered β-turn conformation, exposing more internal hydrophobic regions of the recombinant proteins, enhancing hydrophobic interactions, and increasing S_0_ (Alizadeh-Pasdar and Li-Chan, 2000). The rise in surface hydrophobicity stems from the conformational change of the ELP tag, which promotes hydrophobic interactions between protein molecules. This behavior directly correlates with elevated hydrophobicity, as hydrophobic regions tend to aggregate to minimize their contact with water (Chandler, 2005).

Protein flotation is a separation technique that exploits the differences in protein surface properties to achieve the separation and enrichment of proteins using foam (Han et al., 2020). Foam separation experiments demonstrated that hSODLEH achieved higher Ep, Rp, Ee, Re than hSOD. This confirms that the hydrophobic ELP-fusion protein can be more effectively separated and enriched through foam flotation. Several factors influence protein flotation efficiency, including pH, ionic strength, bubble size, gas flow rate, temperature, protein hydrophobicity (Bergfreund et al., 2021; Chen et al., 2025; Li et al., 2023; Pan et al., 2022; Schwenzfeier et al., 2013; Zhao et al., 2024b). Among those, protein hydrophobicity plays a pivotal role. Hydrophobic proteins have a higher affinity for air-water interfaces as they reduce interfacial energy by displacing water molecules (Damodaran, 2005).

The increased surface hydrophobicity of hSODLEH not only promotes its adsorption at the gas-liquid interface but also strengthens the hydrophobic interactions within the foam matrix (Bergfreund et al., 2021). When adsorbed onto the bubble surface, hSODLEH’s hydrophobic amino acid residues interact with the bubble surface, preventing bubbles from easily rupturing and coalescing. This stability allows the foam to more effectively carry hSODLEH to the solution surface, enhancing the flotation efficiency and enrichment. Hu et al. developed a two-stage foam separation to efficiently recover protein from Perilla seed meal (PSM) using glycine betaine as an enhancer, achieving a total recovery rate of 94.5% and an enrichment factor of 7.1. They demonstrated that glycine betain engaged in cation-π interactions with aromatic residues of PSM protein, which made the protein structure unfold and enabled its hydrophobic groups of PSMP to be more exposed. These factors, plus protein aggregation, were responsible for the enhanced interfacial adsorption of PSMP (Hu et al., 2021).

The increase in surface hydrophobicity caused by the conformational change of the ELP tag also leads to the aggregation of hSODLEH (Wierenga and Gruppen, 2010). Münch et al. demonstrated that diverse ALS-causing mutations provoke SOD1 aggregation by increasing their propensity to expose hydrophobic surfaces. They found that exposure of hydrophobic surfaces precedes aggregation. This shows that aggregation of diverse pathogenic SOD1 mutants is driven by intermolecular hydrophobic interactions either between constitutively hydrophobic mutants or aggregation intermediates exposing hydrophobic surfaces (Münch and Bertolotti, 2010). As quantitatively demonstrated in Table 1, the surface hydrophobicity of hSODLEH increased at elevated temperatures. The enhanced hydrophobicity promotes intermolecular interactions that drive the protein aggregation, as directly evidenced by the increase in hydrodynamic radius (Figures 2C,D) and the formation of large aggregates (Figures 2G,H). These aggregates are more easily captured and carried by foam, further enhancing the separation efficiency of hSODLEH in foam flotation.

ELP is a class of synthetic peptides derived from elastin, composed of the Val-Pro-Gly-Xaa-Gly (VPGXG)n repeat unit, where the guest residue Xaa represents any amino acid except Pro. In this study, valine was selected as the guest residue (Xaa), and repeat number (n) to was set to 50 to enhance the hydrophobicity of the ELP. The decision to use 50 repeats of the VPGVG sequence was based on the fact that the hydrophobicity and thermal responsiveness of ELP are positively correlated with the number of repeats (n). A length of 50 repeats falls within the commonly reported range for ELPs (20–330) and represents a well-characterized and moderate choice that provides a clear thermal transition and sufficient hydrophobicity. Our findings provide a foundation for systematically exploring how key structural parameters of ELP-specifically the repeat number (n) and the identity of the guest residue (Xaa)-affect foam separation performance. Future work could investigate ELP variants with systematically varied repeat lengths (n) and guest residue (Xaa) hydrophobicities to establish a quantitative correlation between ELP architecture and key separation metrics, including enrichment ratio and recovery yield. This approach is anticipated to generate valuable insights that will facilitate the optimization of ELP-based flotation system designs. Additionally, it holds the potential to reveal the fundamental molecular mechanisms governing protein-bubble interactions. Such discoveries could open new and promising directions for developing more efficient, customized ELP-based flotation agents tailored to specific flotation applications.

Protein purification remains a critical challenge for large-scale bioproduction. In this study, the performance of our two-step purification strategy (foam separation + ITC, FM-ITC) was evaluated and compared with other conventional methods for SOD purification, as summarized in Table 3. Previous studies have reported SOD purification using Ni-NTA affinity chromatography from various sources *Cohnella* sp. A01, *Pseudoalteromonas* sp. ANT506, *Halomonas s*p. ANT108, *Homo sapiens*. Additionally, methods such as size exclusion chromatography (e.g., Superdex, Sephadex G-100, Superdex 200, Sephadex G-75) and ion exchange chromatography (e.g., DEAE-Sepharose, DEAE-32, CM-cellulose, Q-Sepharose) have been widely applied across species (Table 3). Our integrated FM-ITC approach achieved a purification fold of 37.52 and a recovery rate of 85.84%, which compares favorably with many multi-step chromatographic methods in terms of both efficiency and overall yield. This strategy also reduces the need for expensive chromatography resins and equipment, simplifies the purification process into two non-chromatographic steps, and presents a scalable alternative suitable for industrial applications. This enhancement arises from the synergistic integration of ELP’s hydrophobicity and thermoresponsiveness: foam separation leverages ELP-mediated hydrophobic adsorption at the gas-liquid interface for initial enrichment, while ITC utilizes temperature-induced phase transitions to further purify the protein.

**TABLE 3:** Comparison of purification efficiency of SOD by different methods.

Source	Protein	Method	Recovery rate (%)	Purificati-on (fold)	Refs
*Homo sapiens*	Cu/ZnSOD	Foam separation	93.32	2.15	This work
*Homo sapiens*	Cu/ZnSOD	ITC	91.98	17.45	This work
*Homo sapiens*	Cu/ZnSOD	Foam separation + ITC	85.84	37.52	This work
*Homo sapiens*	Cu/ZnSOD	One round ITC	79.69	24.27	Wang et al. (2024)
*Homo sapiens*	Cu/ZnSOD	Two round ITC	73.93	26.59	Wang et al. (2024)
*Homo sapiens*	Cu/ZnSOD	Three round ITC	62.33	26.81	Wang et al. (2024)
*Homo sapiens*	Cu/ZnSOD	Ni-NTA	81.4	10.9	Lin et al. (2018)
*Halomonas* sp. ANT108	Cu/ZnSOD	Ni-NTA	43.67	3.61	Wang et al. (2020a)
*Pseudoalteromonas* sp. ANT506	PsSOD	Ni-NTA	22.9	12.6	Wang et al. (2016)
*Cohnella* sp. A01	CaSOD	Ni-NTA	79	2.0	Shahi et al. (2021)
*Homo sapiens*	Cu/ZnSOD	Purified by resilin-like polypeptide-tag	—	24.03	Zhao et al. (2024a)
*Exiguobacterium* s*p.* OS-77	MnSOD	Superdex 200	41	50	Nonaka et al. (2014)
*Marinomonas* sp. NJ522	MnSOD	Sephadex G-75 (2nd)	9	38	Zheng et al. (2006)
*Allium sativum* L	CuZnSOD	Sephacryl S200-HR gel filtration, DEAE Sepharose ion exchange chromatography, chromatofocusing	—	82	Hadji et al. (2007)
*B. licheniformis* SPB-13	Fe/MnSOD	Ammonium sulphate	32.26	1.81	Thakur et al. (2018)
		DEAE-Sepharose	25.16	16.17	Thakur et al. (2018)
*Geobacillus* sp. EPT3	MnSOD	DEAE-Sepharose	14.9	8.0	Zhu et al. (2014)
		Phenyl-Sepharose	11.1	13.4	Zhu et al. (2014)
*L. lactis* M4	MnSOD	Immobilised metalaffinity chromatograph	44.19	1.75	Tan et al. (2014)
		Gel filtration	22.35	3.64	Tan et al. (2014)
*A. gonensis* KA 55 MTCC	SOD	Ammonium sulphate	31.7	2.14	Bhatia et al. (2018)
		DEAE Sephadex A-50 column	12.89	33.11	Bhatia et al. (2018)
*Zizyphus mauritiana* Lamk	SOD	DEAE-fraction	14.8	4.5	Kumar and Malhotra, (2008)
		Sephadex G100 fraction	12.6	12.2	Kumar and Malhotra, (2008)
*Macrobrachium nipponense*	CuZnSOD	DEAE-32	—	12.44	Yao et al. (2007)
		CM-cellulose	—	17.87	Yao et al. (2007)
*Thermothrix* sp	MnSOD	Sephadex G 75	24	4.5	Seatovic et al. (2004)
		Sephadex G 75 + QAE Sephadex	15	105.4	Seatovic et al. (2004)
Human Erythrocyte*s*	CuZnSOD	DEAE-cellulose chromatography + copper chelate affinity chromatography	33.8	196.3	Karadag and Bilgin, (2010)
*Enteromorpha linza*	FeSOD	Q-sepharose FF	28.3	35.6	Lü et al. (2013)
		Q-sepharose FF + Superdex 200	19.1	103.6	Lü et al. (2013)
*Aspergillus glaucus 363*	CuZnSOD	Superdex	35	11.8	Abrashev et al. (2016)
		Superdex + Phenyl-Sepharose	8.1	28.8	Abrashev et al. (2016)
*Aspergillus niger*	CuZnSOD	Anion exchange chromatography	75	—	Hatzinikolaou et al. (1998)
*Curcuma aeruginosa* Roxb	SOD	80% (NH_4_)_2_SO_4_ cut	24.78	0.30	Moon-ai et al. (2012)
		DEAE-cellulose	3.56	1.74	Moon-ai et al. (2012)
		DEAE-cellulose + Sephadex-75	2.51	4.36	Moon-ai et al. (2012)
Tomato fruit	SOD	Ammonium sulphate precipitation + Sephadex G-100 + DEAE-cellulose column chromatographies	44	22	Kumar et al. (2004)

In this study, the purification process was conducted using a sequential approach initiating with foam separation followed by ITC (FM-ITC). The reverse order (ITC followed by foam separation, ITC-FM) were not experimentally evaluated. The FM-ITC order was selected based on the following considerations. First, initiating purification with foam separation allows for efficient capture and concentration of soluble hSODLEH directly from the crude lysate, effectively reducing sample volume and removing significant hydrophilic contaminants. This step provides a pre-enriched feedstock that enhances the subsequent efficiency of ITC. Second, the reverse order (ITC-FM) require resolubilization of the thermally aggregated product prior to foam separation could be applied, introducing additional handling and potential losses. Furthermore, the aggregated form of the protein might exhibit lower adsorption efficiency at the air–water interface, which could adversely affectfoam separation performance. While the FM-ITC sequence demonstrates encouraging efficiency and practical advantages, a systematic comparison of different purification order or single-step represents a valuable direction for future research. Such comparative studies would provide further insight into process optimization and scalability assessment.

It should be pointed out tha the purity of hSODLEH after two-step purification was ∼85%, as assessed by SDS-PAGE (Figure 5B). While this level of purity, achieved in the absence of conventional chromatography, is competitive for many industrial applications such as cosmetics and nutraceuticals, pharma-grade SOD typically requires a higher purity standard (≥95–98%). For applications demanding ultra-high purity, the present method can serve as an efficient initial capture and intermediate purification step. Subsequent polishing steps, such as ion-exchange or size-exclusion chromatography, may be incorporated to further enhance purity to meet pharmaceutical requirements. Additionally, this study was conducted at a laboratory scale (mL to L). Although the results demonstrate a promising purification strategy, further validation through scale-up experiments and process stability studies will be essential to assess its industrial applicability. Notwithstanding these considerations, the method presents several potential practical advantages. By replacing expensive affinity chromatography resins with low-cost consumables such as ammonium sulfate and minimizing the need for complex instrumentation, the process operates on a simpler and potentially more economical basis.

## 5 Conclusion

In this study, a two-step purification strategy integrated foam separation (leveraging ELP’s hydrophobic properties) with ITC (utilizing ELP’s thermoresponsive behavior) to isolate and purify hSODLEH from cell lysate. During the foam separation, the hydrophobic ELP tag promoted selective adsorption of hSODLEH to the gas-liquid interface, enabling efficient concentration and enrichment. Under optimized conditions (protein concentration: 0.4 mg/mL, temperature: 30 °C), the method achieved an Ep of 1.93, Rp of 86.57%, Ee of 2.15, and Re of 93.32%. Subsequently, ITC purification achieved 91.98% recovery rate and 17.45-fold purification fold. Overall the two-step process resulted in 85.84% total recovery rate and 37.52-fold overall purification fold, with a purity of approximately 85%. These results highlight that the dual functionality of ELP-hydrophobicity for interfacial targeting and thermoresponsiveness for phase separation-offer a novel strategy for efficient protein separation and purification. The method not only simplifies the purification workflow and reduces dependence on chromatography, but also offers economic and scalability benefits. Future studies should focus on enhancing protein purity through further process optimization and implementing the strategy on a larger scale to evaluate its feasibility for industrial production. The combination of thermoresponsive and hydrophobic properties of ELPs offers a promising platform for developing sustainable and high-throughput protein purification technologies.

## Data Availability

The raw data supporting the conclusions of this article will be made available by the authors, without undue reservation.
