# Supplementary figures and images for: Two-step purification of elastin-like polypeptide-fusion superoxide dismutase via hydrophobicity and thermoresponsiveness
Source: Front Bioeng Biotechnol. 2025 Oct 24;13:1695586. doi: 10.3389/fbioe.2025.1695586 (PMC12592124; doi:10.3389/fbioe.2025.1695586)

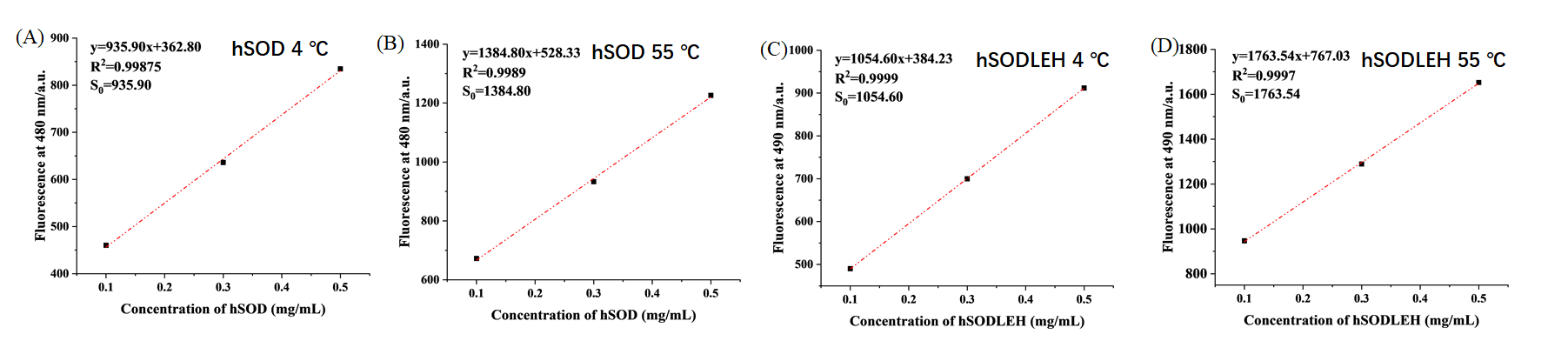


**Figure S1**. Fluorescence intensity of of hSODH and hSODLEH at at 4 and 55 ℃.

Supplement: Supplementary file 1 [file Supplementaryfile1.docx]
